# Identifying Atypical Development: A Role of Day-Care Workers?

**DOI:** 10.1007/s10803-019-04056-3

**Published:** 2019-05-29

**Authors:** Dajie Zhang, Iris Krieber-Tomantschger, Luise Poustka, Herbert Roeyers, Jeff Sigafoos, Sven Bölte, Peter B. Marschik, Christa Einspieler

**Affiliations:** 1 Department of Child and Adolescent Psychiatry, iDN, interdisciplinary Developmental Neuroscience, University Medical Center Goettingen, 37075 Goettingen, Germany; 2 Division of Phoniatrics, iDN, interdisciplinary Developmental Neuroscience, Medical University of Graz, Graz, Austria; 3 Department of Experimental Clinical and Health Psychology, Ghent University, Ghent, Belgium; 4 School of Education, Victoria University of Wellington, Wellington, New Zealand; 5 Center of Neurodevelopmental Disorders (KIND), Center for Psychiatry Research; Department of Women’s and Children’s Health, Karolinska Institutet & Child and Adolescent Psychiatry, Stockholm Health Care Services, Stockholm County Council, Stockholm, Sweden; 6 Curtin Autism Research Group, School of Occupational Therapy, Social Work and Speech Pathology, Curtin University, Perth, WA Australia

**Keywords:** Day-care workers, Developmental disability, Early identification, Retrospective video analysis, Fragile X syndrome

## Abstract

Identifying the early signs of developmental disability is important for ensuring timely diagnosis and early intervention. Day-care workers may be in a prime position to notice potential developmental deviations, but it is unclear if they can accurately recognize subtle early signs of atypical development. Sixty day-care workers examined home-videos of very young children with fragile X syndrome and typically developing children. Results indicated that most day-care workers can distinguish typical and atypical development in general and might therefore have an important role in early identification. Special work experience and advanced pedagogical training appeared to boost day-care workers’ sensitivity to detect atypical features in early development and to provide effective daily surveillance.

Scientific understanding and public awareness of young children at risk for developmental disorders have substantially increased in the past few decades. The growing number of studies on early human development have provided new empirical findings that have altered perspectives on the age-specific phenomena, pathways, and profiles associated with several developmental and genetic disorders (e.g., Johnson et al. 2015; Marschik et al. 2013; Messinger et al. 2013; Thomas et al. 2009). The advancements related to studies on late detected developmental disorders (LDDDs), for example, are particularly evident in research on autism spectrum disorder (ASD; e.g., Bölte et al. 2016, 2013; Bontinck et al. 2018; Bussu et al. 2018; Gliga et al. 2014; Messinger et al. 2013; Roeyers 2018). Research advances have also contributed to what appears to be greater acknowledgment of the significance of earlier detection of atypical development to enable timely intervention and support.

In many European countries, as the number of working-mothers keeps climbing, the need of public childcare services increases steadily. In Austria, for example, the proportion of infants and children under 2 years of age who are attending day-care centres doubled from 2007 to 2017. In some urban areas, up to 45% of children aged 0 to 2-years attend day-care, of whom more than 90% spend 6–10 h per day with professional caregivers (Statistik Austria 2018). These day-care workers could thus be seen as among the most important care persons for many young children. As a consequence, it would seem important to investigate the potential role of day-care workers in the early detection of deviant development in young children. For example, might it be feasible to enlist day-care workers in the process of screening children for ASD? Such issues have been highlighted in a number of recent publications (Branson et al. 2008; Dereu et al. 2012; Janus et al. 2018; Janvier et al. 2016; Larsen et al. 2018a, b; Nordahl-Hansen et al. 2018, 2013).

Indeed, results from some studies suggest that day-care workers have the competency to accurately report early signs of autism by applying well-designed behavioural checklists (Dereu et al. 2010; Larsen et al. 2018b). There might also be some potential advantages in integrating the help of day-care workers because such personnel often have training and knowledge of early child development and have considerable hands-on experience in working with children of comparable ages and diverse developmental profiles. Given that developmental disabilities affect at least 7 to 9% of young children (Olusanya et al. 2018; Zablotsky et al. 2017), it may be important to involve day-care workers as potential screeners of developmental disability. Unlike parents, day-care workers are likely to be more objective. The input from their daily surveillance may complement to our understanding of the prodromal period of LDDDs and potentially contribute to earlier identification, hence be of important public and scientific interest.

Fragile X syndrome (FXS) is one of these LDDDs. Similar to several other disorders, such as Rett syndrome (RTT) and ASD, syndrome related behavioural and physical features are often subtle and elusive to detect at first, which of course makes early identification more challenging. Still, atypical early signs emerging in the first years of life across different developmental domains have been reported to be perceptible to parents of children with FXS (e.g., Hinton et al. 2013; Zhang et al. 2017). Some of these signs are frequently presented and often captured by home videos. With a benchmark procedure retrospectively analysing home videos of children with FXS, we, among other researchers, demonstrated that these signs can be readily identified and classified by professionals (Zhang et al. 2018; see also Baranek et al. 2005). Early phenotypes of FXS present a broad spectrum of atypical neurobehavioural features across various domains (e.g., motor, cognition, speech-language, social-communication), which are not specific to FXS (e.g., Haessler et al. 2016; Hagerman 2002; Kidd et al. 2014; Marschik et al. 2014; Raspa et al. 2017; Roche et al. 2018; Zhang et al. 2017). As early signs of FXS overlap with peculiarities of children with other developmental and genetic disorders, to identify these signs requires sensitivity to deviant development in general and not necessarily expertise in a specific syndrome.

Pertinent to the current study, we intended to utilize home-video footage showing behaviours of different developmental areas of typically developing children and children with FXS as material to tap on day-care workers’ general awareness of early development. We adapted a video reviewing procedure used by Burford et al. (2003) by adding benchmark assessments of neurodevelopmental features. Specifically, we aimed to investigate whether day-care workers perceive young children with typical or atypical development differently. In Addition, we intended to find out whether they are able to accurately identify typical and atypical features in early development. As day-care workers have different training and experience backgrounds, we also aimed to answer whether these factors modify day-care workers’ perceptions of early developmental phenomena.

## Methods

### Participants

Twelve day-care centres and one vocational school for kindergarten teachers in Graz, Austria and surrounding regions were contacted. Seven centres with 36 day-care workers (21 kindergarten teachers, one male; 15 carers, all female) and the school with 25 pre-service student teachers (all female) agreed to participate. Of the 21 teachers, 8 received additional advanced pedagogical or special-needs training. The professional training for kindergarten teachers in Austria is 5 years, and for carers 6 months. None of the 15 carers in this study had received additional training related to childcare. At the time of data collection, the mean age of teachers was 42 years (SD = 11.40), and that of the carers was 43 years (SD = 11.50). The 25 pre-service student teachers had recently finished the penultimate year of their training, and were on average 17 years of age (range 17–19). Their curriculum in the penultimate year includes 2 h supervised hands-on training per week at day care centres. One pre-service student teacher dropped out due to personal reasons, leaving the final sample with a total of 60 participants (21 teachers, 15 carers, 24 pre-service student teachers).

In addition to levels of training, we collected data of work experiences of the participants, including the overall length, and whether the participant had professional experience with children younger than 2 years of age and/or with a developmental disorder.

## Materials

This Citizen Scientist project is linked to an umbrella project on early development of individuals with FXS (Zhang et al. 2017). For the current study, we extracted data from our real-world database GUARDIAN (Graz University Audiovisual Research Database for the Interdisciplinary Analysis of Neurodevelopment). We selected recordings of 13 children with FXS and 7 typically developing children (TD), of whom videos of the first 24 months of life were available. All children with FXS had received a clinical and genetic diagnosis, and all the videos were recorded prior to diagnosis. All TD children were singletons, born at term with uneventful pregnancy and delivery. At the time of this manuscript preparation, all were older than 7 years of age, none has received a diagnosis of any developmental or genetic disorder, nor did any of their known family relatives. A total of 35 (rounded) hours recordings (25 h from the children with FXS), including footage of daily routines or special family gatherings, were first cut into clips by a research assistant. Clips were cut by settings, which were defined by changes of the child’s position (e.g., in prone/supine, sitting in rocker, walking with support) and the presence/absence of the caregiver(s) in the scene; for technical details on clipping please see Zappella et al. (2015). Following the aims of this study, we initially selected 40 clips of the FXS corpus and 20 of the TD corpus covering different age bands of the first 2 years of life (1–6, 7–12, 13–18, and 19–24 months). The clips were chosen to present behaviours in different developmental areas (e.g., motor, social-communication) of the target child. After a pilot trial and intramural testing, we retained 15 clips to trim the experiment duration (please see data collection below), leaving 10 videos from children with FXS (FXS videos from here after) and 5 from TD children (TD videos from here after). These 5 TD videos were comparable to 5 of the 10 FXS videos in age and setting (Table 1). All clips had a length of 1 min (± 5 s).

**Table 1:** Benchmark features for the FXS and TD videos

	Video code^a^	Age (months)	Areas with features on the benchmark list^b^							**n/60**
			**Mot**	**Phy**	**RM**	**S/C**				
						Fac	Int	Lan	Voc	
FXS videos	FXS1	8	++		+	+			+	17
	FXS2	9		+	+	++	+		+	13
	FXS3	9	++	+				+	+	15
	FXS4	10	++			++	+	+		7
	FXS5	10	+		++		+			9
	FXS6	12	++		++	+	+	+		6
	FXS7	12	+			++				26
	FXS8	13	+		+		++		+	6
	FXS9	16	+		+	++		+		7
	FXS10	23	+		+		+	++	+	21
TD videos	TD1	8	+		+		+	++		31
	TD2	9	+		++		+		+	48
	TD3	10	+						++	46
	TD4	16	++				+	++		9
	TD5	23					++	+		48

**++**This area consists of the primary feature defined for this video on the benchmark list. For three videos (FXS4, FXS6, and TD4), two primary features have been identified

**+**This area consists of non-primary features identified for this video

**n/60**: Number of participants regarded the child as normal

The benchmark list includes age-specific features (in alphabetical order) in four areas (in bold): **Mot** (motor): age-advanced/positive features (coasting/sideward walking, fine manipulation, standing up independently, good postural control), age-inadequate features (no antigravity movements, no manipulation, not sitting without support, not standing-up free, not walking free, tapping objects without grasping), and in addition, slumped posture/hypotonia, long-lasting tongue protrusion; **Phy** (physical appearance): oversized ears, strabismus; **RM** (repetitive movements): body rocking, hand/arm flapping, hand opening and closing, head shaking; **S/C** (social/communication) with (a) Fac (facial expression): awkward smile, crying-like facial expression during pleasure vocalizations, empty gaze, non-adaptive facial expression, sluggish facial expression; (b) Int (interaction): positive features (attentive and patient, engaged in play, interactive and responsive) and negative features (exaggerated reaction, lack of adaptive reaction in social play, no response when spoken to, passive/no initiative in social play, roughness with other children); (c) Lan (language): age-inadequate utterances, neologisms; (d) Voc (vocalizations): expiratory and inspiratory vocalizations, high-pitched vocalizations, hoarse voice, monotonous/repetitive vocalizations, echolalia, pressed voice, unmodulated vocalizations

^a^Videos from children with neurotypical outcome, TD1 to TD5, were comparable to FXS1, FXS2, FXS4, FXS9, and FXS10, respectively, in age and setting. Videos are ordered by ascending age (completed months)

^b^Multiple features were frequently identified in the same area for a video

The present study was approved by the Institutional Review Board of the Medical University of Graz, Austria (29-054 ex 16/17). Participants and parents who shared their data gave their informed written consent to participate in the study and to publication of the results.

### Data Collection

Video reviewing took place for each participant individually in a separate quiet room at the kindergarten or school where the participant worked or was trained. The participants were given background information about the study and instructed to look for early developmental markers that might contribute to timely identification of late-diagnosed developmental disorders. After signing the informed written consent, the participants were asked to review the 15 video clips from young children between 8 and 23 months of age. They were also informed that some of the children were typically developing, and some were later diagnosed with a developmental disorder. The term “fragile X syndrome” was not mentioned. The participants were encouraged to comment on each video based on their experience with similar aged children while taking into account the different developmental domains. The order of presenting the 15 video clips was randomized across participants. Before playing each video, the participant was informed of the child’s gender and age, but not whether the child had received a diagnosis. Each video was played twice on a computer, controlled by an experimenter. The participant was encouraged to take notes after the first run. After the second run, the participant was asked to comment on anything worthy of attention about the child, both positive notes and concerns. The participant was never requested to judge normal versus atypical development. The next clip was played when the participant was ready. Pauses were made when necessary, but not during the same clip. The review of all 15 clips took about 60 min for each participant. An experimenter guided the review procedure, took notes, and ran the computer program without engaging in any discussion on the participant’s comments. On-screen audio recording was taken by Corel VideoStudio X9, using the “Screen Capture” function.

### Transcription and Scoring

All 60 audio recordings were later transcribed verbatim in the iDN lab by the author I.K.T. About 15% of the audio materials were randomly selected and transcribed by another research assistant to check on the accuracy and consistency for the scoring of the transcriptions. No meaningful difference has been revealed. Thus, all scoring was solely based on the transcripts from I.K.T.

Three senior experienced team members of the research group iDN (D.Z., P.B.M., and C.E.) reviewed the 15 video clips without knowing the medical backgrounds of the children. They separately outlined all features worthy of attention for each target child, being particularly positive or age-advanced (e.g., coasting/sideward walking at 10 months of age) as well as atypical or age-inadequate (i.e., referring to delayed developmental milestones and/or abnormal behaviours). The features covered different neurofunctions of various developmental areas (e.g., motor, social, verbal, physical appearance, repetitive movements). Although typical and age-appropriate behaviours (e.g., sitting without support) were observed in the clips of children with FXS, none was pointed out as remarkable or age-advanced. Thus, no particularly positive or age-advanced feature was listed for any of the FXS videos. For TD videos, both positive and atypical features were listed. The three authors also named one or two “primary feature(s)” for each clip, referring to the most salient feature(s) observed in the scene. They then discussed their analyses and reached consensus on a benchmark list for each clip. Each list consists of at least three features besides the primary one. Multiple features were frequently identified in the same developmental area (e.g., motor, social) for one video. For two clips from children with FXS and one from a TD child, two primary features were identified (Table 1).

To assess the comments of the participants, we implemented a scoring scheme adapting the optimality concept introduced by Prechtl (1980) and the inductive corpus analytical methodology of Lindseth and Norberg (2004). Higher scores indicate greater congruity with the benchmark list. Each comment made on a clip was assigned a score between 0 and 7; details on scoring are given in Table 2.

**Table 2:** Scoring scheme for FXS and TD videos

Scale for comments on FXS videos		Scale for comments on TD videos	
Raw score	Definition	Raw score	Definition
7	Correct identification of 3 or more features including the primary feature	7	Correct identification of 2 or more features including the primary feature
6	Correct identification of 2 features including the primary feature	6	Correct identification of the primary feature or multiple none-primary features
5	Correct identification of the primary feature or multiple none-primary features	5	Correct identification of a none-primary feature
4	Correct identification of a none-primary feature	4	No identification of any relevant feature, but making feature-related comments
3	No identification of any feature, but making feature-related comments^a^	3	Finds nothing worthy of attention
2	No irrelevant remarks^b^	2	No irrelevant remarks
1	No opposite interpretation of the features^c^	1	No opposite interpretation of the features
0	No concern	0	Only opposite interpretations of the features

Opposite interpretation leads to point deduction. For example, if a participant correctly identified the primary feature of an infant with FXS, yet referred to the child’s another atypical feature as normal (i.e., opposite interpretation), the score for this clip would be 4 points (5 points, 1 point deduction)

^a^For example, a feature on the benchmark list of a video was “empty gaze”. A participant commented “He looks somehow strange to me. I wonder if he could see at all? Maybe he is blind?”

^b^The participant makes comments traceable to the scene, but fails to identify any feature on the benchmark list

^c^Opposite interpretation: an obviously atypical behaviour seen as normal, or vice versa

In the next step, D.Z. and I.K.T. separately assessed all 60 transcripts. Intraclass correlation coefficient (ICC) between the scorers was .94 (95% CI, .90–.97), based on the model of average measures, absolute-agreement, and two-way mixed-effects. Discrepancies were then discussed until consensus was reached. In addition, we counted for each video the number of participants who commented that the target child was normal or expressed no concern about the child’s development (Table 1).

### Data Analysis

Data were analysed with SPSS, version 25 (SPSS Inc, Chicago, IL). Mann–Whitney U test was run to compare the difference of two independent groups if normal distributions could not be assumed. *T* test was used to compare the difference between two means of normal distributions. Pearson correlation coefficient (r) was calculated to estimate the association of two continuous variables with normal distributions. Spearman rank order correlation (rho) was applied to estimate the association between two variables if normal distributions could not be assumed for both. ANOVA was run to compare means among three or more independent groups. Eta squared (η^**2**^) was chosen to report the effect size of U test and ANOVA. Cohen’s d was reported for the effect size for t-test. Alpha was set to .05, two-tailed for all analyses.

## Results

### Normal Versus Atypical Development?

More participants commented that the target child was developing normally for TD videos (Md = 46 out of 60, range 9–48; Table 1) compared to FXS videos (Md = 11, range 6–26), Mann–Whitney U = 5.50, Z = 2.40, p = .013, with large effect size η^**2**^= .38. For all but one TD video, more than half of the participants spontaneously remarked that the target child was normally developing. This video showed a child of 16 months of age, who did not produce linguistic vocalizations, nor walked or stood up independently on the clip.

### Consistency Between Participants and the Benchmark Analysis

Initial analysis of the mean raw scores of the 5 TD videos as well as the 10 FXS videos revealed normal distributions. The mean raw score for the TD videos across participants was 4.04 (SD = 1.24) and 3.13 (SD = .93) for the FXS videos. The standard mean raw score of TD (Z_TD_) and FXS videos (Z_FXS_) was significantly correlated, r = .43, p = .001. That is, participants who scored high (or low) on the TD videos were also likely to do so on the FXS videos. The overall score of a participant is the standard sum of Z_TD_ and Z_FXS_, i.e., overall score = Z [Z_TD_ + Z_FXS_]. The overall score ranged from − 2.23 to 2.26 across participants. A higher overall score of a participant indicates higher consistency with the benchmark analysis of the videos. We examined the following variables:

#### Training Status

The overall scores differed significantly among teachers with or without additional pedagogical or special-needs training (st; teachers^**st+**^ and teachers^**st−**^ from here after), carers, and pre-service student teachers, F_(3, 56)_ = 5.45, p = .002, η^**2**^= .23. A Tukey posthoc comparison revealed significantly higher overall scores for teachers^**st+**^ (M = 1.15, SD = .96) compared to teachers^**st−**^ (M = .05, SD = .92, d = 1.17, p = .043), to carers (M = − .18, SD = .99, d = 1.36, p = .008), and to pre-service student teachers (M = − .30, SD = .82, d = 1.62, p = .001). No statistically significant difference of the overall score between any of the two groups was found among teachers^**st−**^, carers, or pre-service student teachers.

#### Work Experience

The pre-service student teachers were excluded from this analysis as they had not yet started to work on a daily basis. The length of work experience of the 34 day-care workers^1^ was not normally distributed. The median was 7.5 years (range 2–34). Of the 20 teachers, the median was 7.5 years (range 2–34); of the 14 carers, it was 8.0 years (range 2–19). The difference was not significant, Mann–Whitney U = 136.00, Z = .141, p = .888. The overall scores of the day-care workers correlated moderately with the length of their work experience, Spearman’s rho = .34 (p = .052). No significant difference of the overall score was found between participants with longer (i.e., length of experience above median, n = 16; overall score M = .51, SD = .96) or shorter (n = 18, overall score M = .08, SD = 1.03) work experiences, Student’s independent sample t-test yielded t = 1.26, p = .218.

#### Task Relevant Experience

Task relevant experience requires: (a) a reasonable amount of general work experience as a day-care worker (i.e. length of work experience being at least 5 years), and, (b) has professional experience working with young children (i.e., under 2-year-old) and/or (c) children with a developmental disorder. Twelve teachers and 6 carers met the above conditions and were classified as the group as having “rich” task relevant experience, including all of the 8 teachers^**st+**^. The mean overall scores of the “rich” group was .68 (SD = .94). The remaining 8 teachers and 8 carers who had never worked with young children nor children with a developmental disorder, or, who had less than 5 years of professional experience as a day-care worker were taken into the “limited” task relevant experience group. The mean of their overall score was -.16 (SD = .92). The difference of the overall score between the “rich” and “limited” groups was significant, t = 2.65, p = .012, d = .90. That is, participants with rich task relevant experiences had higher scores. Further analysis revealed that teachers with rich task relevant experience (M = .98, SD = .89) scored higher than teachers with limited task relevant experience (M = − .21, SD = .95), t = 2.86, d = 1.29, p = .010. Scores of the carers with rich (M = .08, SD = .75) or limited (M = − .11, SD = .95) task relevant experience, however, were not significantly different, t = .41, p = .688. In other words, task relevant experience influenced the scores of the teachers, but not the carers.

## Discussion

The present study, based on our previous research and methodologically similar studies (Burford et al. 2003; Dereu et al. 2012; Marschik et al. 2012; Zhang et al. 2017, 2018), presents a novel approach for investigating day-care workers’ awareness on early development and the prodromal phase of late recognized developmental disorders. We tapped on the participants’ views and knowledge of early developmental milestones and qualitative deviances from normal development during the first 2 years of life. Although participants were not explicitly asked to differentiate between normal and atypical development, their comments indicated that they clearly perceived differences between TD and children with deviant development, FXS in this case. Significantly less concerns but more spontaneous confirmation of normality were addressed to TD compared to children with FXS. That is, normal or aberrant development in general was perceptible to the day-care workers.

This finding is similar to those of Marschik et al. (2012) in a study that focused on acoustic Gestalt perception. They found that both speech-language pathologists and non-professionals were able to distinguish typical vocalizations from atypical vocalizations expressed by young children. In the current study, most of the participants noticed that *something* was wrong when they were observing a child who was later diagnosed with FXS, but when it came to specifying exactly *what* was wrong, that was where differences among the different participant groups emerged. Specifically, observations from teachers^**st+**^ (i.e., with additional specialized trainings in atypical development) were most consistent with the benchmark assessments of developmental scientists. Teachers^**st−**^ (i.e. without additional training) performed less well and comparable to the pre-service student teachers in the penultimate year of training, and, again similar to the carers (i.e., with much shorter professional training). This, not surprisingly, suggests that specialized training beyond the general childcare preparation may underpin the sensitivity to recognize typical and atypical features in early development (see also Daniels and Mandell 2014; Lee et al. 2005; Reilly et al. 2015).

Given these findings, it leaves open the question concerning what, if any, role does work experience play? Interestingly, teachers and carers who had worked longer in day care units did not score significantly better than those with shorter work experience. This was echoed by the findings that teachers^**st−**^ and the pre-service student teachers performed rather similarly, both having comparable level of training, while the teachers having practiced longer. In other words, the duration of experience did not prove to be critical to developing “sensitive eyes” for assessing early developmental phenomena. Rather, the *profile* of experience seemed to play a vital role in this respect. In our current study, it was not childcare experience per se that mattered, but rather having “task-relevant experience”, that is, having practical experience with certain groups of children (e.g., children younger than 2 years and/or children with a developmental disability) that was associated with higher scores. Still, it must be pointed out that the task-relevant experience appeared to have an impact only if a solid training basis was already present. That is, only the teachers, but not the carers, seemed to have benefited from the task-relevant experience in that they performed better compared to their counterparts with less experience. This suggests that without sufficient general training, accumulating experience alone might not actually induce better insight into children’s early development.

Incidentally, yet not unexpectedly, all 8 teachers^**st+**^ fell into the group of 16 participants with rich task-relevant experience. These eight teachers who pursued advanced trainings had also accordantly practiced their know-how in their daily work. Both specialized training and related practices may therefore have accounted for their performance in recognizing typical versus atypical features in early development. For day-care workers who intensively interact with children of different ages and developmental profiles day in and day out, to notice that *something* may be wrong with a child’s development might be unchallenging. However, in order to more precisely specify developmental anomalies that may be indicative of a developmental disability, specialized training and rich experiences seem to be critical factors.

Notably, the raw scores assessed according to the scoring scales (Table 2) indicated that many of the teachers^**st−**^, the carers, and the pre-service student teachers failed to recognize any specific feature listed by the benchmark assessment for most video sequences (i.e., having scored on average lower than 4 for the FXS videos and lower than 5 for the TD videos). The features which were salient to the professionals who set the benchmarks appeared to have escaped the eyes of many of the participants. This finding would seem to call into question the reliability of asking day-care workers to detect specific warning signs of a possible developmental disability in very young children. Similarly, studies focusing on older children revealed that teachers’ agreement with other informants on reporting developmental phenomena varied across different contexts (Ehlers et al. 1999; Macari et al. 2018; Mattila et al. 2009; Nordahl-Hansen et al. 2013, 2014; Posserud et al. 2006). In contrast, a previous study demonstrated that specialist nurses with extensive experience in home and hospital surveillance of infants were well able to detect early signs of developmental disability (i.e., RTT) and distinguish the infants from typical developing controls (Burford et al. 2003). This highlights the potential significance of qualification both in training and practice. The findings are also in line with our present study in that those day-care workers with advanced training and experience were better able to identify early typical and atypical developmental features. Another study on a large cohort by Dereu et al. (2010) used a specially developed checklist for childcare workers to screen children at risk for ASD. After a targeted short training highlighting early signs of ASD and how to use the checklist, the childcare workers were proven to be reliable and sensitive informants of early signs of ASD. This and our present study together suggest that day-care workers have the potential to distinguish typical and atypical development in general. To gain most from day-care workers’ input to support early identification of atypical development, using specifically designed checklist of signs and providing related training may be effective and desirable.

We are well aware that our study brings along some limitations. The experimental design using brief video sequences does not fully resemble natural settings, where day-care workers may be able to observe each child across a range of contexts over days, weeks, and months. Moreover, some of our participants might not have felt comfortable in an experimental situation, which could have negatively influenced their performance. Nonetheless, reviewing videos provides a sound approximation of the real world setting and can deliver valuable information within a reasonable time. As all the participants had the same materials to assess, distinctions among groups might be attributed to their different sensitivities to various developmental features. It is rather unlikely that simply providing more extensive and relaxed observational settings will lead to significantly better insights into normalities and peculiarities in early development. In their daily work field, day-care workers might be reluctant to alarm parents or healthcare professionals about atypical development of a child due to ethical and personal reasons, even if they would have noticed adverse signs. Thus, researchers and healthcare professionals need to actively collect data in an efficient way from qualified day-care workers if they intend to profit from additional critical information which they cannot gain from parents (see also Garcia-Primo et al. 2014; Macari et al. 2018). Also, the small sample size is another limitation related to the present work.

Last but not least, investigations into the early signs of LDDDs have mostly been done with retrospective approaches, including utilizing questionnaires and checklists, as well as analyses of home videos. This has been gradually overtaken by prospective approaches for a number of disorders, ASD being at the forefront (e.g., Bölte et al. 2013; Isaksson et al. 2018; Loth et al. 2016; Messinger et al. 2013; Ozonoff et al. 2015). Due to well-discussed limitations as well as benefits of both approaches (e.g., Marschik and Einspieler 2011; Ozonoff et al. 2011; Palomo et al. 2006), the prospective methods will unlikely fully replace the retrospective ones. Especially when studying rare diseases and disorders such as RTT, mainly caused by de novo mutations of the *MECP2* gene, for which obtaining high-risk samples is unrealistic (Marschik et al. 2018), retrospective procedures are still of great value.

## Conclusion

Day-care workers are not trained healthcare professionals and it is not their responsibility to identify children with an atypical development. Rather, with their intensive daily interaction with young children of different developmental profiles, they might be among the first to notice aberrant development in very young children. Our healthcare system might benefit most from day-care workers by allocating staff members with advanced training and experience in every childcare institution, who will facilitate daily developmental surveillance and may help healthcare professionals to identify otherwise late recognized developmental disorders at a younger age.

## Footnotes

1 For one of the 21 teachers and one of the 15 carers, data of the length of work experience was missing, leaving 20 teachers and 14 carers for the comparison.
